# Two-Stage Surgery Using FROZENIX Partial ET for Frozen Elephant Trunk Technique and Open Descending Aortic Replacement in a Patient With Recurrent Type B Aortic Dissection and Microscopic Polyangiitis: A Case Report

**DOI:** 10.7759/cureus.67055

**Published:** 2024-08-17

**Authors:** Ryoji Kinoshita, Taiju Watanabe, Ryumon Matsumoto, Kazunobu Hirooka

**Affiliations:** 1 Cardiovascular Surgery, Tsuchiura Kyodo General Hospital, Tsuchiura, JPN; 2 Cardiac Surgery, Tsuchiura Kyodo General Hospital, Tsuchiura, JPN

**Keywords:** two-stage surgery, descending thoracic aortic aneurysm, frozen elephant trunk, microscopic polyangiitis (mpa), type b aortic dissection

## Abstract

The frozen elephant trunk (FET) technique, initially developed as a one-stage procedure to treat extensive thoracic aortic aneurysms, has since been adapted to address acute and chronic aortic dissections by closing entry tears and expanding the true lumen. It has become widely adopted due to its effectiveness in managing aortic diseases. We present the case of a 39-year-old female with microscopic polyangiitis (MPA) who developed recurrent type B aortic dissection accompanied by rapid expansion. The patient, a compromised host with multiple comorbidities such as glomerulonephritis, chronic renal failure, alveolar hemorrhage, and acute pancreatitis, required urgent surgical intervention. Given the complexity of her condition and the high risks associated with direct surgery, a staged approach was selected. The first stage involved using a novel FET prosthesis, the FROZENIX Partial ET (FPET), inserted via median sternotomy, followed by a left thoracotomy for non-deep hypothermic circulatory arrest (non-DHCA) descending aortic replacement. The surgery led to favorable outcomes without any major complications or sequelae.

FPET offers distinct advantages in this complex scenario. Its design features a 2 cm stent-free distal section, which reduces the risk of distal stent graft-induced new entries (dSINEs) and simplifies anastomosis during the second stage of surgery. For patients with severe comorbidities and anatomical challenges that make the thoracic endovascular aortic repair (TEVAR) unsuitable, a staged open surgical approach is a viable alternative, mitigating the risks linked to DHCA. This case underscores the utility of a staged surgical approach using FPET in managing complicated chronic type B aortic dissection in patients with significant comorbidities. The FPET prosthesis facilitates effective lesion control while minimizing the risk of dSINEs and streamlining subsequent surgical procedures, presenting a promising strategy for similar complex cases.

## Introduction

The frozen elephant trunk (FET) technique was initially proposed as a one-stage curative surgery for extensive thoracic aortic aneurysms [1]. However, since its inception, the scope of FET has expanded to include not only entry closure but also the expansion of the true lumen in both acute and chronic aortic dissections. The technique has demonstrated effectiveness in a range of aortic pathologies and has become widely adopted for the treatment of aortic diseases [2].

In this report, we present the complex case of a 39-year-old woman diagnosed with microscopic polyangiitis (MPA), a condition associated with glomerulonephritis, chronic renal failure, alveolar hemorrhage, and acute pancreatitis, among other complications. This patient experienced a recurrent type B aortic dissection that showed rapid expansion, necessitating prompt surgical intervention. To manage this high-risk case, we employed a staged surgical approach using a novel FET prosthesis, the FROZENIX Partial ET (FPET; Japan Lifeline, Tokyo, Japan), as the initial procedure. Through this report, we aim to demonstrate the utility of a two-stage surgery utilizing FPET in treating complicated chronic type B aortic dissection in a patient with multiple comorbidities.

## Case presentation

The patient was a 39-year-old female with a significant medical history. Nine years ago, she had been diagnosed with MPA based on high levels of myeloperoxidase-anti-neutrophil cytoplasmic antibody (MPO-ANCA) and kidney biopsy findings. This condition had led to necrotizing glomerulonephritis, causing progressive renal failure, and she had started maintenance dialysis for end-stage renal disease seven years ago. She had also experienced alveolar hemorrhage as a complication of MPA and drug-induced acute pancreatitis due to cyclosporine, which had been used to control inflammation.

**Figure 1:**
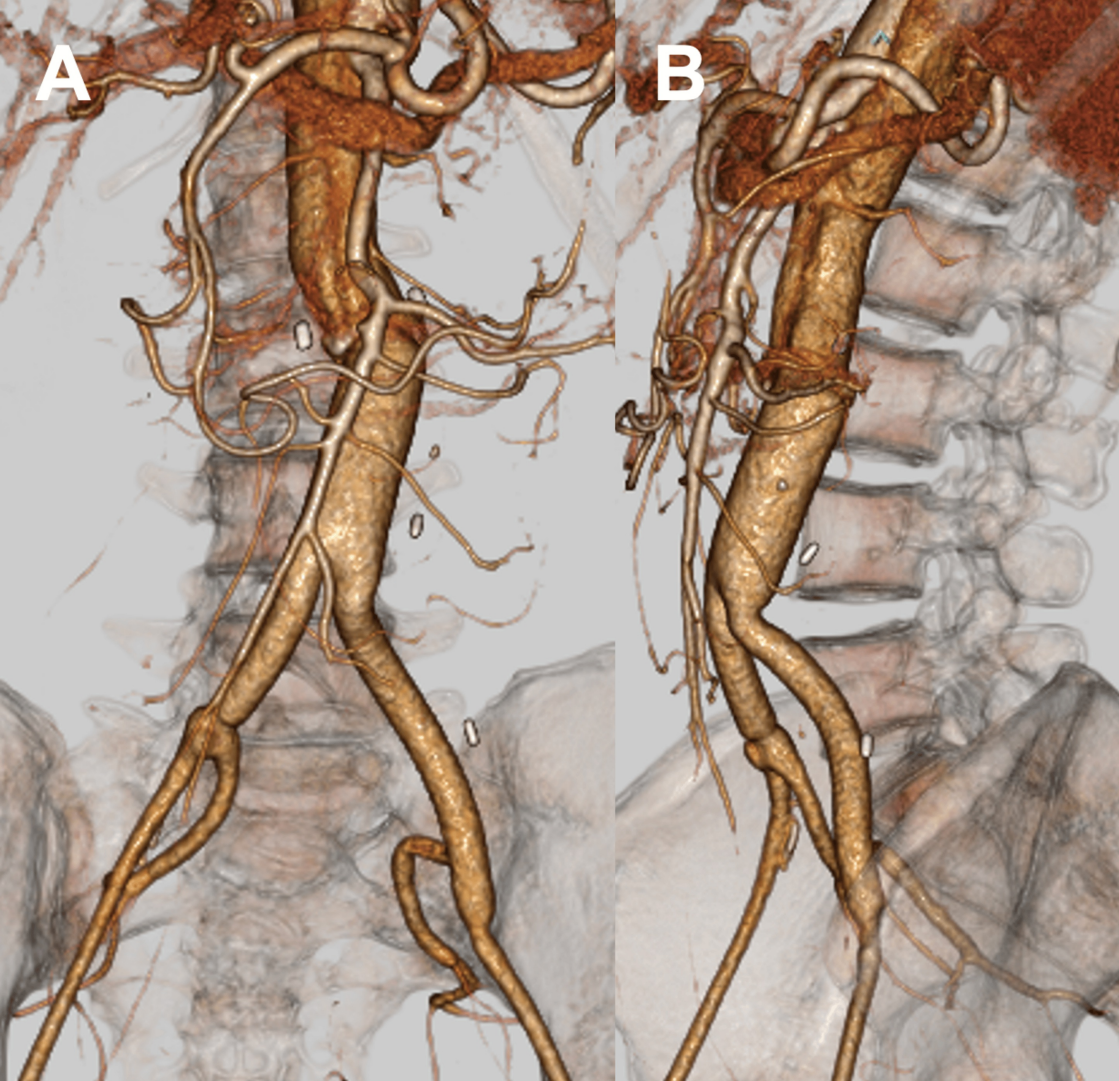
3D CT images after Y-graft replacement with a double-barrel anastomosis technique A. 3D CT image in the frontal view. B. 3D CT image in the left anterior oblique (LAO) view CT: computed tomography

Her history of aortic dissection had begun five years ago with an acute Stanford type B aortic dissection, which extended from the distal arch just distal to the left subclavian artery bifurcation to both common iliac arteries. Conservative management with medication had been undertaken, and she had been subsequently monitored through imaging studies. Over time, the left common iliac artery aneurysm had shown signs of enlargement, reaching a maximum diameter of 38 mm approximately two years ago (three years after the initial dissection), leading to surgical intervention. She had undergone left common iliac artery aneurysm resection and infrarenal aortic Y-graft replacement (J Graft 18×9 mm; Japan Lifeline), with the proximal aorta anastomosed using a double-barrel technique (Figure 1).

**Figure 2:**
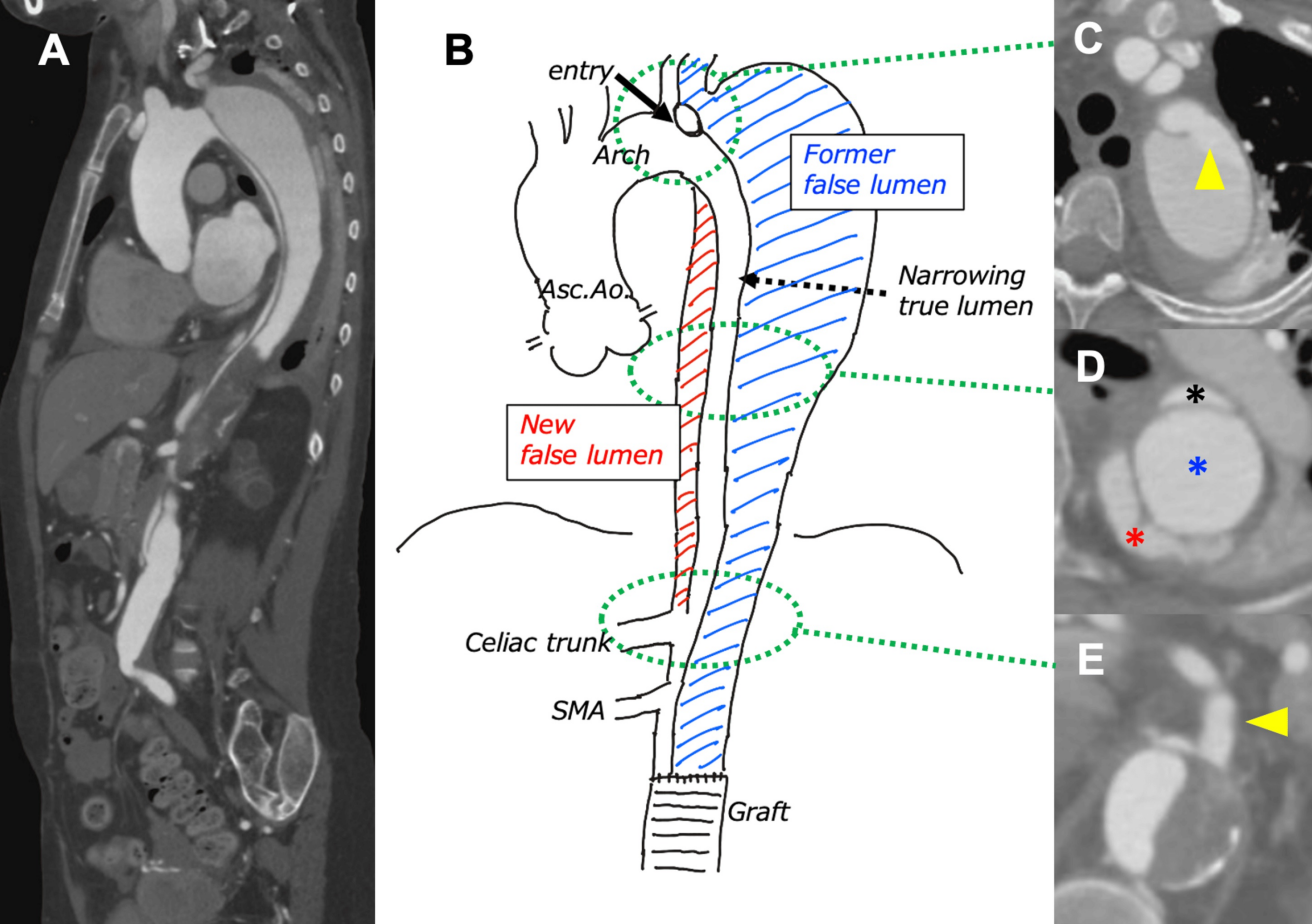
CT images and schematic of recurrent type B acute aortic dissection with triple-barrel dissection A. Sagittal image of the entire thoracic and abdominal region. B. The schematic of the patient’s type B aortic dissection shows the relationship between the narrowing true lumen, former false lumen, and new false lumen. C. An entry into the former false lumen (yellow arrow) is observed just distal to the left subclavian artery branch. D. A newly formed false lumen is observed on the dorsal side of the descending aorta, resulting in a triple-barrel dissection (black asterisk: narrowing true lumen, blue asterisk: former false lumen, red asterisk: new false lumen). E. The new false lumen communicated with the true lumen through a fenestration at the celiac trunk (yellow arrow). This illustration is an original creation by the authors CT: computed tomography

Following this, she had been under observation again until she experienced sudden back pain, prompting an emergency visit to a nearby hospital. Contrast-enhanced CT at the time had revealed a narrowed true lumen (22 mm in the major axis and 7 mm in the minor axis), an expanded false lumen, and a newly formed false lumen, creating a triple-barrel dissection (Figure 2) and indicating recurrent acute Stanford type B aortic dissection. 

**Figure 3:**
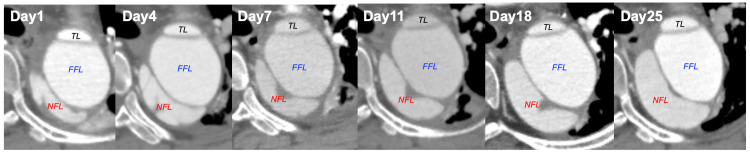
Rapid progressive changes in aortic dissection Due to the rapid expansion of the newly formed false lumen, the aortic diameter increased from 45 mm at admission to 55 mm three weeks later TL: true lumen; FFL: former false lumen; NFL: new false lumen

She was then transferred to our hospital, where we initiated conservative treatment with strict blood pressure control in the ICU. Despite rigorous blood pressure management, she experienced recurring back pain, and imaging studies showed a rapid expansion of a new dissection cavity in the dorsal descending aorta. The maximum diameter of the entire aortic aneurysm increased from 45 mm at admission to 55 mm over approximately three weeks (Figure 3), necessitating early surgical intervention. Echocardiography also revealed moderate aortic regurgitation. At this point, her MPA was maintained with no recurrence of inflammation while on a regimen of multiple immunosuppressants, including oral prednisolone 10 mg, mizoribine 50 mg, and azathioprine 25 mg.

In summary, the patient presented with pain that was difficult to control and a rapidly expanding complicated type B acute aortic dissection, compounded by multiple comorbidities. While minimally invasive thoracic endovascular aortic repair (TEVAR) was considered to avoid the risks of open surgery, anatomical challenges made entry closure difficult, and concerns about long-term outcomes persisted due to the patient’s young age and underlying vasculitis. Thus, this option was dismissed. Open surgery was deemed the only viable treatment. Hence, in this case of type B aortic dissection without lesions in the ascending aorta, a one-stage left thoracotomy with prosthetic vascular graft replacement was considered.

However, management for proximal anastomosis in this scenario would require deep hypothermic circulatory arrest (DHCA), which posed a high risk due to the patient’s history of alveolar hemorrhage and moderate aortic regurgitation. Therefore, there was concern that the surgical invasion would be excessively severe. Therefore, we aimed to manage the surgical risk by performing a staged procedure, involving an elephant trunk insertion via median sternotomy followed by graft replacement under aortic cross-clamping via left thoracotomy, which does not require deep hypothermic management. Approximately six weeks after the recurrent Stanford type B acute aortic dissection, we performed the first surgery through median sternotomy. After establishing cardiopulmonary bypass, we managed moderate hypothermia with a target deep body temperature of 28°C, employing antegrade cerebral perfusion and lower body circulatory arrest.

**Figure 4:**
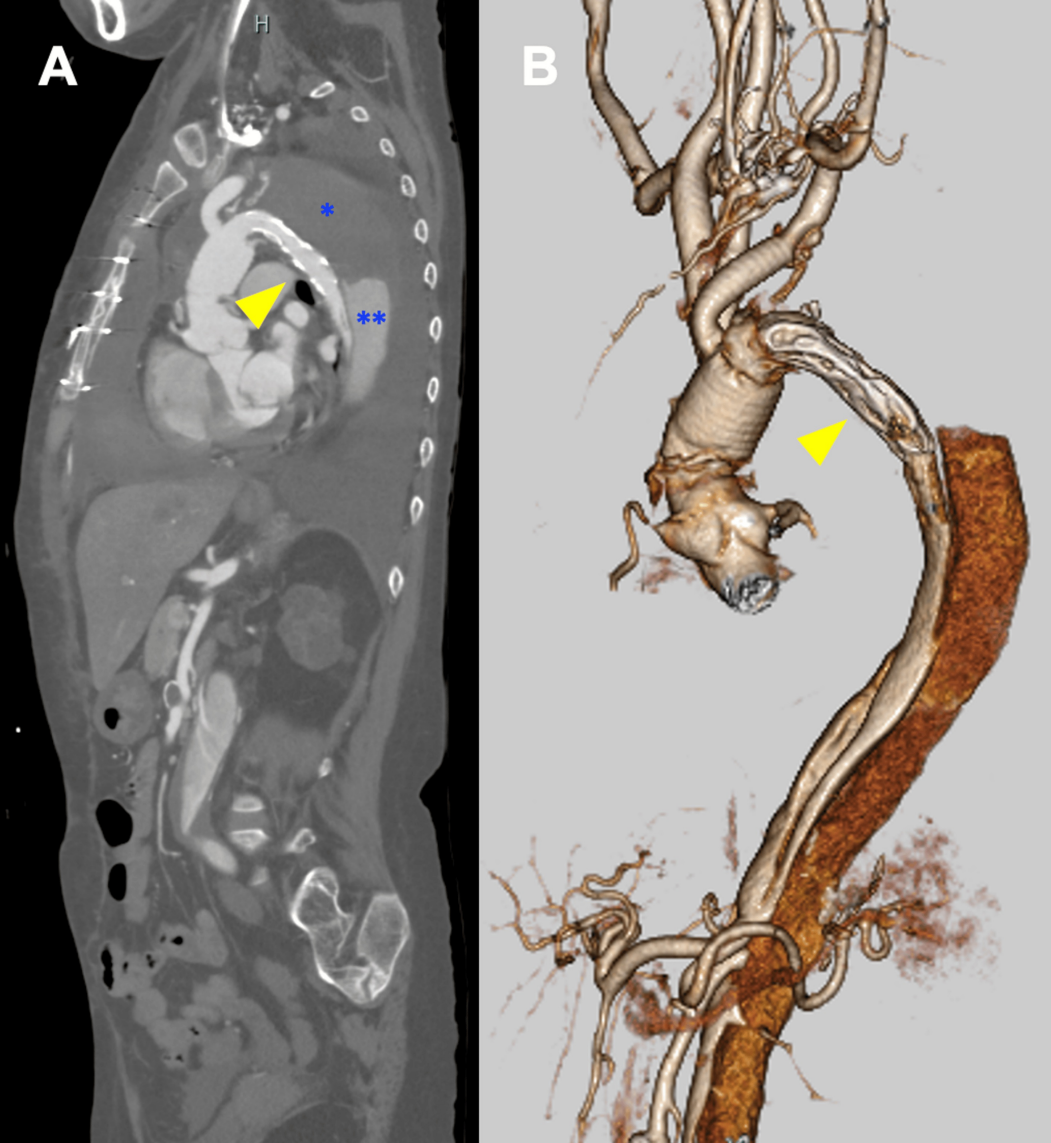
CT images after the initial surgery A. Sagittal image. B. 3D CT image. Thrombosis of the former false lumen is observed in the proximal descending aorta (blue asterisk), while contrast enhancement from distal re-entry is seen beyond the mid-descending aorta (double blue asterisk). Despite the insertion of a stent graft as part of the FET, sufficient expansion of the true lumen (yellow arrow) was not achieved CT: computed tomography; FET: frozen elephant trunk

Under circulatory arrest, we deployed a J Graft FPET Open Stent Graft 25 mm (Japan Lifeline) as a frozen elephant trunk in the true lumen of the distal arch aorta. We then replaced the entire aortic arch using a 24 mm four-branched graft (J-Graft spiral 4 branch; Japan Lifeline). Regarding the moderate aortic regurgitation, it was caused by slight prolapse due to elongation of the free margin of the left coronary cusp; hence, we performed a central plication with 6-0 prolene, improving the regurgitation to a mild grade, and concluded the surgery (Figure 4). Postoperatively, the patient developed acute pancreatitis, requiring fasting and medication; her condition eventually improved with this treatment alone, and she was discharged ambulatory on postoperative day 24. After allowing time for postoperative recovery, we performed the second stage of the surgery approximately 10 weeks after the first.

**Figure 5:**
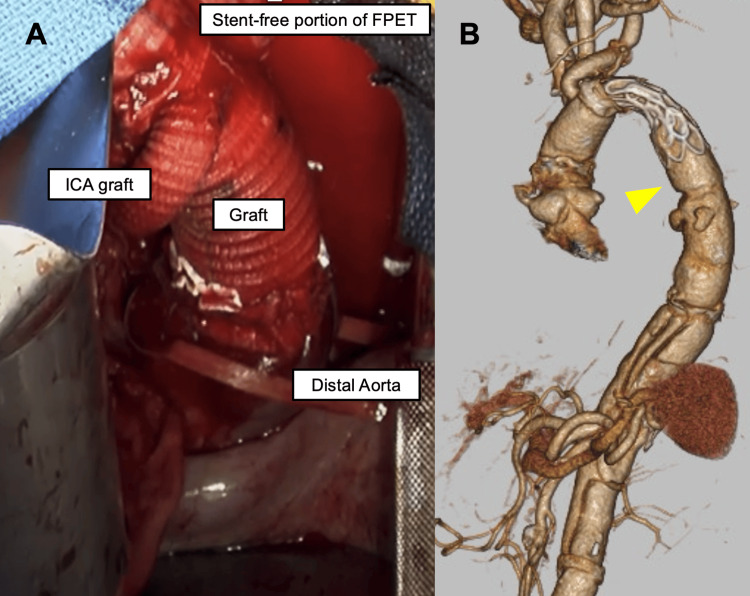
Final result after reconstruction with two-stage surgery A. Intraoperative findings during the second stage left thoracotomy. B. 3D CT image showing the entire aorta after reconstruction. The stent-free portion below the stent frame is the distal end of the FROZENIX Partial ET graft (yellow arrow) CT: computed tomography; FPET: FROZENIX Partial ET; ICA: intercostal artery

Using a left thoracotomy approach in the right lateral decubitus position, we exposed the descending aorta. Epiaortic echo clearly identified the position of the frozen elephant trunk inserted during the initial surgery (Video 1). Under cardiopulmonary bypass assistance via femoral cannulation, we clamped the proximal descending aorta, including the entire frozen elephant trunk. After opening the aorta distal to the clamped site and exposing the distal end of the frozen elephant trunk, we re-clamped only the stent graft and utilized the stent-free portion at the distal end of FPET for anastomosis to the distal descending aorta graft (J-Graft straight 26mm; Japan Lifeline). To prevent paraplegia, we reconstructed the intercostal arteries and, due to the risk of pancreatitis recurrence, avoided abdominal manipulation by anastomosing the distal descending aorta within the thoracic area. The intimal septum in the distal aorta, which had formed a triple-barrel configuration, was resected as much as possible under aortic clamping, and the graft was anastomosed at this site (Figure 5).

**Video 1:** Intraoperative epiaortic echo The source of this video is attributed to the authors

After the second stage of surgery, the patient showed rapid overall improvement without significant complications and was discharged ambulatory on postoperative day 15. During the perioperative period of both surgeries, there were no significant neurological abnormalities or major infectious complications. She is currently under outpatient follow-up, maintaining a good overall condition.

## Discussion

MPA is a type of ANCA-associated vasculitis characterized by necrotizing vasculitis affecting small vessels, such as capillaries, venules, and arterioles, with a high positive rate for ANCA. The necrotizing vasculitis of systemic small arteries can cause hemorrhage or infarction in major organs, leading to complications such as interstitial pneumonia and alveolar hemorrhage in the lungs, and progressing to necrotizing glomerulonephritis to the point of requiring dialysis in the kidneys [3]. While vasculitis does not generally occur in the aorta or its major branches, cases of aortic disease related to ANCA-associated vasculitis have been reported [4-7]. In our case, it is unclear whether there is a causal relationship between the aortic dissection and microscopic polyangiitis; however, given the occurrence of multiple aortic dissections in a young 39-year-old woman, along with episodes suggesting large vessel fragility, there may be some involvement. The wide variety of patient symptoms and illnesses associated with Stanford type B aortic dissection, as well as the disparate standards of treatment across various healthcare practitioners, make choosing a definitive therapy for Stanford type B aortic dissection a challenging issue.

The definitive treatment for type B aortic dissection varies depending on whether the stage is acute or chronic, and whether there are severe complications during the acute stage. Ultimately, the choice is between endovascular treatment or open surgery. As in our case, the persistent, recurrent pain and rapid expansion identifies the condition as a complicated type B acute aortic dissection. Endovascular treatment is recommended for this patient group due to its superior outcomes in the acute phase [8]. The therapeutic efficacy of TEVAR in aortic dissection lies in its ability to achieve entry closure, blocking blood flow into the false lumen, and expanding the true lumen with the device. This approach prevents the rupture of the weakened false lumen wall and resolves malperfusion of critical organ branches, thus improving acute-phase outcomes. However, there are many cases, like the present one, where anatomical challenges make TEVAR difficult to perform. In young patients and cases where long-term outcomes of endovascular treatment are concerning due to the potential need for reintervention [9], open surgery may be considered.

This case presented significant challenges due to the chronic nature of the aortic dissection, despite it being an acute recurrence. The true lumen was severely narrowed, making standard stent graft deployment risky due to potential intimal damage. Also, the new false lumen entry was unrelated to the typical stent graft closure site, raising doubts about the efficacy of endovascular therapy. Given these anatomical challenges and the patient’s young age, an open surgical approach was deemed more appropriate, as long-term outcomes with TEVAR in chronic dissection cases are often unstable, requiring frequent secondary interventions. Many reports suggest that for type B aortic dissection, especially in the chronic phase, open surgical repair is the standard approach as it offers superior long-term outcomes compared to endovascular treatment [10,11]. However, open surgery for type B aortic dissection remains problematic due to the high risk of perioperative mortality or major complications, especially when using DHCA, which has been linked to a surgical mortality rate of 28% [12].

In cases of type B dissection, where there are no lesions in the ascending aorta, a left thoracotomy is used to approach the descending aorta and perform direct graft replacement distal to the descending aorta. However, when the chronic aortic dissection extends to the distal arch or the entry tear is near the distal arch, proximal aortic clamping in left thoracotomy surgery for type B aortic dissection is often difficult or risky. Aortic clamping carries the risk of retrograde type A dissection, a potentially fatal complication. Thus, performing proximal anastomosis under circulatory arrest is often necessary in surgeries via left thoracotomy for aortic dissection. DHCA offers advantages such as minimizing mobilization of the proximal aorta, eliminating risks associated with aortic clamping, and providing organ protection, particularly neuroprotection, due to hypothermia. However, this technique involves significant perioperative risks, including complications from deep hypothermia such as coagulopathy, myocardial injury due to ischemia during ventricular fibrillation, and pulmonary hemorrhage associated with coagulopathy [13].

In this case, there was a history of alveolar hemorrhage due to MPA, raising significant concerns about the risk of alveolar hemorrhage associated with performing DHCA. Once alveolar hemorrhage occurs, managing the bleeding becomes challenging due to hypothermia and full heparinization during cardiopulmonary bypass. Also, it leads to decreased oxygenation and ventilation capabilities, making it difficult to maintain stable respiratory management with a ventilator alone. This makes the risk of alveolar hemorrhage a clear major perioperative predictor of poor outcomes. Furthermore, moderate aortic regurgitation posed a risk of myocardial ischemia due to ventricular fibrillation during hypothermia, which also made the implementation of DHCA challenging. Even if the aortic arch could be clamped with antegrade cerebral perfusion, the risk of retrograde type A dissection in a case with such recurrent aortic dissection made it clear that a one-stage surgery via left thoracotomy would entail excessively high perioperative risks, rendering it practically infeasible.

In the treatment of type B aortic dissection, where DHCA poses high risks, performing an initial replacement of the ascending aorta and arch followed by staged surgery on the descending aorta can effectively control lesions from the distal arch to the proximal descending aorta. This approach is well-known as the elephant trunk procedure, designed for two-stage surgeries. The elephant trunk method was first described by Borst et al. in 1983 as the first-stage surgery for extensive thoracoabdominal aortic aneurysms [14]. Subsequently, Crawford et al. [15] and Svensson [16] acknowledged its usefulness, and it has since become widely adopted. The advantage of the elephant trunk procedure is that, by dividing the surgery into two stages, it reduces hypothermic circulatory arrest time, distributing surgical risks and reducing invasiveness.

Later, a device combining a stent frame with the elephant trunk graft was developed. This aimed to exclude extensive thoracic aortic aneurysms in a single stage by promoting thrombosis on the distal anastomosis side using a stent graft, leading to the development of the FET method, which was reported by Kato et al. in 1996 [1]. Today, commercial devices are manufactured and sold, and the technique is widely used. Over 12,000 prostheses have been implanted since the 2014 launch of the J graft Frozenix® (Japan LifeLine) FET prosthetic, which is handmade in Japan [17].

While the FET prosthesis is a promising device for the treatment of aortic diseases, it is associated with a specific complication called distal stent graft-induced new entries (dSINEs), which arise when the stent frame exerts stress on the aortic intima [18]. To overcome this issue, a new and improved version of the device called FPET, which was also used in our case, was released in 2023. This device includes a 2 cm section without a stent frame at its distal end, consisting only of a vascular graft. This design prevents the stent edge from directly contacting the aortic intima, thus potentially inhibiting the occurrence of dSINEs. Additionally, this 2 cm stent-free section facilitates easier anastomosis with the graft of the distal aorta during the second stage of surgery, which is another attractive feature.

Inserting a conventional elephant trunk accurately into a severely narrowed true lumen, such as in chronic type B aortic dissection, is not an easy task. this can result in being unable to insert a vascular graft at a sufficient distance into the distal true lumen, or, if a long graft is forced into the narrow true lumen, it may lead to bending or twisting of the graft. This, in turn, can fail the safe control and clamping of the elephant trunk graft during the second-stage surgery, defeating the purpose of reducing risk with staged surgery. However, with the FET prosthesis, the radial force of the stent graft frame allows the elephant trunk to maintain its circular shape even within a narrowed true lumen. This ensures it can be placed straight without kinking inside the aorta, and the distal end of the elephant trunk can be positioned as planned preoperatively. Additionally, by using epiaortic echo, as we did, the exact position of the stent graft within the aorta can be clearly identified, enabling guided clamping of the entire aorta. This ensures that the elephant trunk graft can be securely clamped, and the graft end can be safely accessed after an aortic incision.

Using this method eliminates the need for hypothermic management during surgical procedures distal to the descending aorta, significantly reducing surgical risks. In this case, despite the patient having many comorbidities, we were able to provide treatment without any major complications or sequelae.

## Conclusions

We presented a case of the patient, a compromised host due to MPA, glomerulonephritis, chronic renal failure, alveolar hemorrhage, acute pancreatitis, and several other complications, who developed recurrent type B aortic dissection with rapid enlargement. We employed a staged surgical approach using the FET technique with FPET, followed by a left thoracotomy for non-DHCA descending aortic replacement, yielding good results. Despite the benefits of TEVAR, an open chest approach was chosen due to the severe narrowing of the true lumen and the atypical false lumen entry, which posed significant risks that TEVAR could not adequately address. The open surgery enabled better management of these challenges, reducing the risk of complications like dSINE and ensuring long-term stability.

In the treatment of chronic type B aortic dissection, where performing DHCA is challenging, a staged surgical strategy involving the initial insertion of the elephant trunk via median sternotomy, followed by surgery beyond the descending aorta through the left thoracotomy, may be considered. The FROZENIX Partial ET is extremely useful due to its reduced risk of developing dSINEs, a complication specific to FET prostheses, and its facilitation of easy anastomosis at the stent-free distal end during the second stage of surgery.
